# HOW MULTIPLE CHRONIC DISEASE BURDEN INFLUENCES COMMUNITY-BASED BEHAVIORAL HEALTH PROGRAM PARTICIPANTS

**DOI:** 10.1093/geroni/igz038.969

**Published:** 2019-11-08

**Authors:** Keith W Turner, Cheng Yin

**Affiliations:** THE UNIVERSITY OF NORTH TEXAS, DENTON, Texas, United States

## Abstract

Background: Studies have shown that participation in community-based self-management education programs can result in improved healthful behaviors (exercise, cognitive symptom management, coping, and communications with physicians), improved health status (self-reported health, fatigue, disability, social/role activities, and health distress), and decreased days in the hospital. Problem: One of the understudied factors thought to influence efficacy in community based self-management programs is the presence and impacts of multiple chronic conditions on participants within community based behavioral health program populations. Multiple chronic diseases when scaled collectively can be considered as a participant’s individual disease burden to be included in other analyses. Methodology: This investigation explores possible ways disease burden associates with such important constructs as participant personal characteristics and participant confidence in controlling impacts of their disease symptoms and participant preferences for use of various methods of coping with disease impacts. Outcomes: Results indicate a complex pattern of relationships between such factors as personal characteristics of program participants and their perceived mastery over the impacts of their disease symptoms, and their preferred mechanisms for coping. Implications: program designers and managers can better understand the differential influences of disease burden on participants analyzed with their personal characteristics and their preferential uses for coping mechanisms and their perceived ability to withstand the added burdens of multiple chronic diseases.

